# Health Behaviors Among Family Caregivers Participating in the Paid Workforce

**DOI:** 10.1093/geroni/igaa057.1656

**Published:** 2020-12-16

**Authors:** Abiola Keller, Mauricio Garnier-Villarreal

**Affiliations:** Marquette University, Milwaukee, Wisconsin, United States

## Abstract

Up to 20% of family caregivers are also working full-time or part-time. The competing demands of caregiving and working may adversely affect the health of caregivers. In this study, we use data from the 2015 Behavioral Risk Factor Surveillance System Questionnaire-Caregiver module to examine the relationships between participation in the paid workforce and health behaviors among caregivers. Individuals with a positive response to the survey question, “During the past 30 days, did you provide regular care or assistance to a friend or family member who has a health problem or disability?” were identified as caregivers. Participation in the paid workforce was defined as being employed for wages or self-employed. We conducted separate survey-weighted multivariate regression analyses to examine the relationship between participation in the paid workforce and drinking, exercise, and smoking controlling for sociodemographic characteristics. For each outcome, all sociodemographic characteristic variables were entered into the model simultaneously. 55.6% of the 24,034 caregivers participated in the paid workforce. The survey-adjusted multivariate regression analyses showed that those in the paid workforce had an increased likelihood of binge drinking (OR = 1.39, 95% CI [1.14-1.69]) and decreased likelihood of engaging in leisure-time physical activity (OR = 0.83, 95% CI [0.73-0.95]) compared to those who were not in the paid workforce. The odds of smoking less frequently did not differ between the two groups (OR = 0.96, 95% CI [0.85-1.07]). Our findings underscore the need for workplace-based interventions aimed at promoting healthy behaviors among caregivers.

